# *Osodendron* gen. nov. (Leguminosae, Caesalpinioideae), a new genus of mimosoid legumes of tropical Africa

**DOI:** 10.3897/phytokeys.205.82821

**Published:** 2022-08-22

**Authors:** Erik J. M. Koenen

**Affiliations:** 1 Evolutionary Biology & Ecology, Université Libre de Bruxelles, Faculté des Sciences, Campus du Solbosch - CP 160/12, Avenue F.D. Roosevelt, 50, 1050 Brussels, Belgium Université Libre de Bruxelles Bruxelles Belgium

**Keywords:** *Albizia*, *Cathormion*, ingoid clade, Fabaceae, *Samanea*, taxonomy

## Abstract

The genus *Osodendron* is here newly described to accommodate three species and one subspecies of African mimosoid legumes. These taxa have previously been included by several authors in *Albizia*, *Cathormion* and/or *Samanea*, but they have been shown to be phylogenetically unrelated to any of these, being instead the sister-group of the recently described Neotropical genus *Robrichia*, which is similar in vegetative morphology and especially its very similar indumentum, but is decidedly different in pod morphology. A taxonomic treatment of the three species in the genus is presented, with species descriptions, photographs, distribution maps and an identification key. The type species *Osodendronaltissimum* (Hook. f.) E.J.M. Koenen occurs in swamp and riverine rainforest and gallery forests, with the typical subsp. altissimum widespread across tropical Africa, while Osodendronaltissimumsubsp.busiraensis (G.C.C. Gilbert) E.J.M. Koenen is only known from the Busira river catchment in the western part of the Democratic Republic of Congo. Of the other two species, *Osodendrondinklagei* (Harms) E.J.M. Koenen is a common tree of rainforest and the forest-savannah transition including semi-deciduous and secondary forest as well as gallery forest and is restricted to Upper Guinea and the similar, but vegetatively more variable *Osodendronleptophyllum* (Harms) E.J.M. Koenen occupies comparable vegetation types in Lower Guinea and extends marginally into the Sudanian and Zambezian savannahs in gallery forest.

## Introduction

The taxonomy and classification of the mimosoid legumes has seen significant changes over the last 35 years, including the disintegration of *Acacia* Mill. (Pedley 1986; Luckow et al. 2003; Miller and Seigler 2012), multiple contentious reclassifications of the ingoid mimosoids by several authors (e.g. Nielsen 1981; Barneby and Grimes 1996, 1997; Lewis and Rico-Arce 2005; reviewed by Brown 2008), as well as the clade not being recognised as a subfamily (LPWG 2017) anymore, being instead subsumed into a recircumscribed Caesalpinioideae. Following phylogenetic (Luckow et al. 2003; Bouchenak-Khelladi et al. 2010) and phylogenomic (Koenen et al. 2020; Ringelberg et al. 2022) studies, several further changes have recently been proposed (e.g. Souza et al. 2013; Soares et al. 2021).

In all this turmoil, a number of African species that have variously been placed in *Albizia* Durazz., *Cathormion* Hassk. and/or *Samanea* Merr., have been somewhat neglected by most authors. While Barneby and Grimes (1996) mentioned the similarities of some of these species to South-American taxa and Nielsen (1992) excluded them from *Cathormion*, they have not been formally dealt with and, therefore, Lewis and Rico-Arce (2005) referred them to the ‘dustbin’ genus *Albizia* (see Koenen et al. 2020) pending further studies on that genus. This was clearly unsatisfying and botanists working on the African flora have continued treating these species in *Cathormion* and *Samanea* (e.g. Hawthorne and Jongkind 2006: see note on p. 860), but this is an equally poor solution. The phylogenomic studies of Koenen et al. (2020) and Ringelberg et al. (2022) have elucidated the evolutionary relationships of these species for the first time. First, the type species of *Cathormion* was shown to be nested in *Albizia* and the generic name has been synonymised with that genus by Koenen et al. (2020), while the African *Cathormionobliquifoliolatum* (De Wild.) G.C.C. Gilbert & Boutique and *Cathormionrhombifolium* (Benth.) Keay were shown to be closely related to South American *Hydrochorea* Barneby & J.W. Grimes and are transferred to that genus by Soares et al. (2022). This leaves us with three species, *Cathormionaltissimum* (Hook. f.) Hutch. & Dandy, *Samaneadinklagei* (Harms) Keay and *Samanealeptophylla* (Harms) Brenan & Brummit that were placed in the Inga clade by Koenen et al. (2020), but which cannot be placed decisively in any existing mimosoid genus. These three species have since been resolved by Ringelberg et al. (2022) as the sister-group to the recently established South and Central American genus *Robrichia* (Barneby & J.W. Grimes) A.R.M. Luz & E.R. Souza (syn. Enterolobiumsect.Robrichia Barneby & J.W. Grimes; Souza et al. 2022).

**Figure 1.**
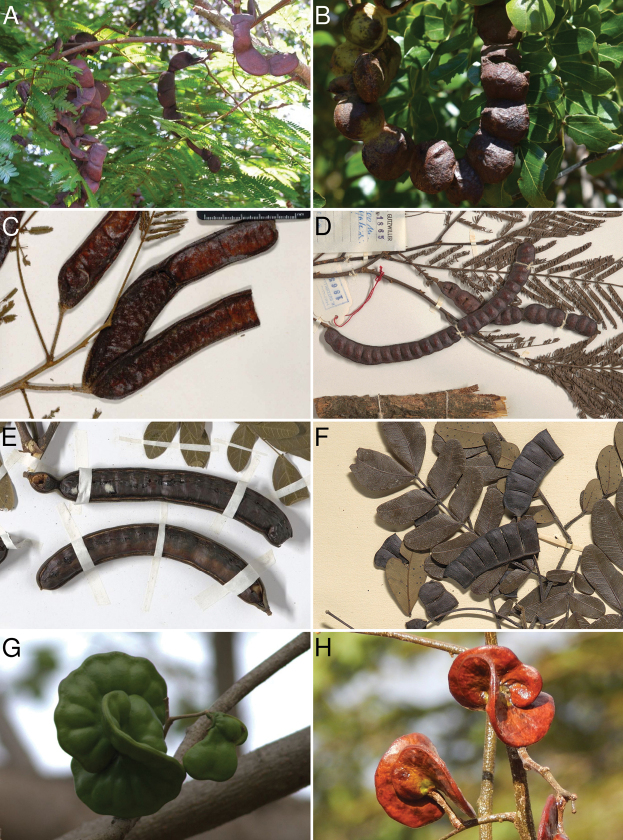
Confusing pods of *Cathormion* s.l. **A** pods of *Osodendronaltissimum* that are very similar to **B** pods of the Australian species *Albiziamoniliformis* (DC.) F. Muell. (syn. Cathormionumbellatumsubsp.moniliforme (DC.) Brummit) **C** pods of *Osodendrondinklagei* and **D** pods of *Osodendronleptophyllum* that were likened to those of **E***Samanea* (*S.saman* (Jacq.) Merr. is pictured here) and/or *Cathormion***F** pods of *Hydrochoreaobliquifoliolata* (De Wild.) E.J.M. Koenen (syn. *Cathormionobliquifoliolatum*) that are superficially similar to those of *O.leptophyllum* in particular; hence, this species having also been classified in a broadly circumscribed *Cathormion* in the past, but note that the pods become disarticulated while still on the tree rather than tardily as in *Osodendron* and *Albiziamoniliformis***G** and **H** pods of the closest relative of *Osodendron*, the Neotropical genus *Robrichia* (*R.schomburgkii* is pictured here). Images: **A** Jos Stevens, voucher unknown **B** Russel Cumming, voucher unknown **C***J. Alves Pereira 2664* (MA) **D***R. Gutzwiller 1865* (P) **E***J.F. Maxwell 91-300* (L) **F***B. Descoings 8278* (MPU) **G** Cristoph Moning, voucher unknown **H** José Benito Quezada Bonilla, *J.B. Quezada Bonilla 623* (HULE, MO). **A** from www.africanplants.senckenberg.de**B–H** from www.gbif.org**B–G** distributed under a Creative Commons BY-NC-SA 4.0 Licence **H** distributed under a Creative Commons BY-NC-SA 3.0 Licence.

**Figure 2.**
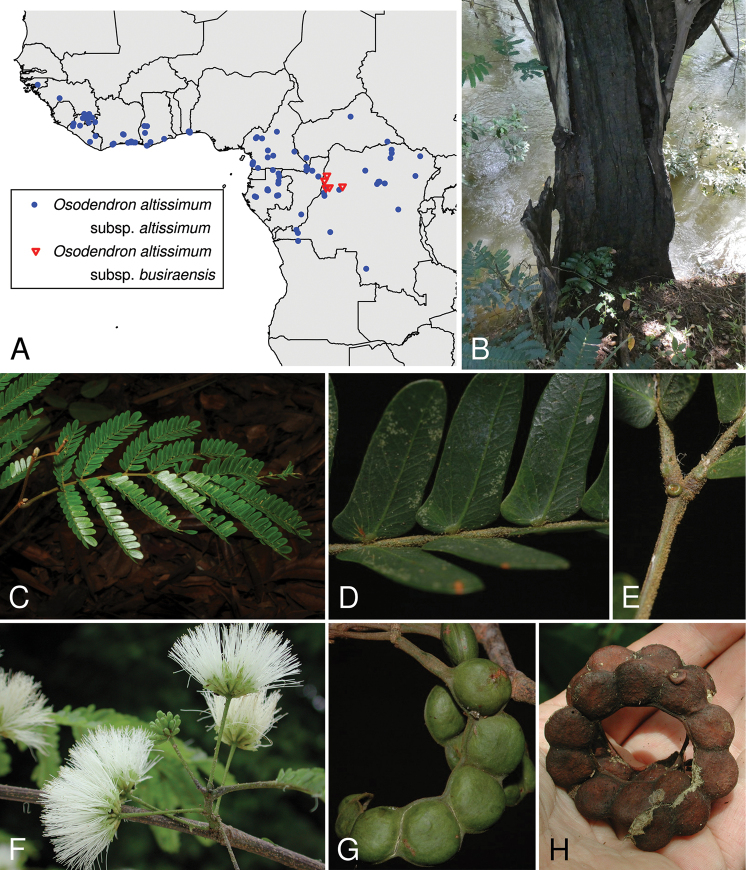
*Osodendronaltissimum***A** distribution of the two subspecies as per the legend, based on data from Gbif.org with the addition of occurrence records for O.altissimumsubsp.busiraensis from specimens at BR **B** trunk on riverbank **C** bipinnate leaf **D** close-up of leaflets **E** close-up of rachis apex showing nectary **F** inflorescences **G** unripe pod **H** mature pod. Images: **B, F, H** William Hawthorne (FHO) **C–E, G** David Harris (E), from www.africanplants.senckenberg.de.

**Figure 3.**
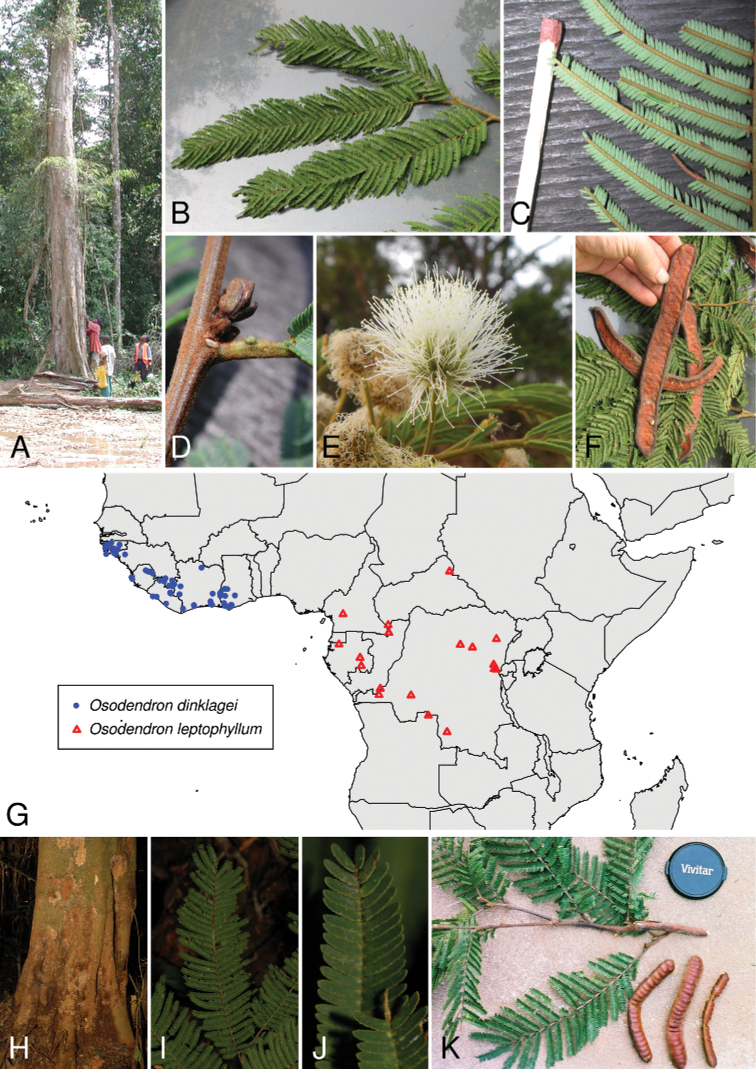
**A–F***Osodendrondinklagei*: **A** trunk of adult specimen in terra firme forest **B** foliage **C** detail of pinnae **D** detail of resting bud and petiolar nectary **E** inflorescence **F** pods **G** distribution map of *O.dinklagei* and *O.leptophyllum* as per the legend based on data from Gbif.org**H–K***O.leptophyllum*: **H** trunk **I** bipinnate leaf **J** close-up of leaflets **K** twig and pods. Images: **A–D, F** William Hawthorne (FHO) **E** Xander van der Burgt (K) **H–J** David Harris (E) **K** Paul Latham **H–K** from www.africanplants.senckenberg.de.

While these African species are clearly similar to *Robrichia* in sharing the same ferruginous pubescence on young parts, as well as having very similar inflorescence morphology, the African species display wider variation in leaf size, leaflet number and shape, but differ most notably in pod morphology. The species of *Robrichia* are characterised by contorted (“ear-shaped”) indehiscent pods (Fig. 1G, H; Souza et al. 2022: Fig. 2D, E, J) which led them to be previously classified in *Enterolobium* Mart., while the related African species have twisted to coiled articulated lomentiform pods (*C.altissimum*, Figs 1A, 2G, H), straight to slightly curved woody pods (*S.dinklagei*, Figs 1C, 3F) or intermediate straight to slightly curved articulated pods (*S.leptophylla*/*C.eriorhachis*, Figs 1D, 3K). The woody pods were likened to *Samanea* and the articulated pods are superficially similar to *Cathormion* s.s.; hence, these species have been placed in either or both these genera in the past.

Given the similarities and sister-group relationship with *Robrichia*, one could argue that these species might be accommodated in that genus, perhaps as a separate section. However, the generic description of *Robrichia* would then have to be considerably expanded to include the greater variation in leaf morphology and especially to encompass the different pod types. Given that the pods of *Robrichia* are highly characteristic, adding these African taxa to that genus would be undesirable and these two groups are equally conveniently accommodated in two separate genera. Therefore, I establish the new genus *Osodendron* E.J.M. Koenen to accommodate these three African species.

Much confusion about relationships and generic classification in mimosoids (especially ingoids) has been caused by homoplasious fruit characters (Ringelberg et al. 2022) and the lomentiform pod type is one of the most intriguing because it has apparently evolved multiple times associated with repeated adaptations to periodically inundated areas and/or riparian habitats to facilitate hydrochorous seed dispersal (Aviles Peraza et al. 2022; Ringelberg et al. 2022; Soares et al. 2022). Indeed, when comparing the pods of *Osodendron* spp. to those of some *Albizia* species (Fig. 1B) or *Hydrochorea* (Fig. 1F), one can understand why these have all been previously included in a broadly circumscribed *Cathormion* and why a close relationship between *Osodendron* and *Robrichia* was not suspected (Fig. 1G, H), even though Barneby and Grimes (1996: p. 247) noted that *Robrichiaschomburgkii* (Benth.) A.R.M. Luz & E.R. Souza and *Samaneadinklagei* are very similar and essentially only differ in their fruits. To aid understanding of the differences in fruit morphology amongst the genera that are either closely related to *Osodendron* or with which these species were thought to share affinities, the critical pod characters of these groups are summarised globally in Table 1. It is clear that *Osodendron* can be separated from the continental African species of *Albizia* and *Hydrochorea* by at least some of the features of the fruit, especially dehiscence, but it becomes more complicated when taking Madagascan, Asian, Australian and Pacific *Albizia* into account. Further studies are required to understand the morphological differences between *Albizia* and its allies and segregates, but it is clear that the genus *Albizia*, under its current circumscription, with the inclusion of *Cathormion* s.s. and exclusion of the New World species (these being transferred to *Pseudalbizzia* Britton & Rose by Aviles Peraza et al. 2022) is monophyletic (Koenen et al. 2020; Ringelberg et al. 2022; Koenen et al. unpubl. data). Regardless of this wider global context, *Osodendron* can be readily separated from all other ingoid genera on the African continent, for which I include an identification key below.

**Table 1.** Fruit characters of *Osodendron* and morphologically similar genera.

	fruit shape	pod valve texture	septate between seeds	dehiscence
*Osodendron* – Africa	straight, slightly curved, coiled and/or twisted	woody, papery to thinly ligneous or aerenchymous	yes	indehiscent or tardily breaking up into articles
*Robrichia* – Neotropics	contorted/ “ear-shaped”	woody	yes	indehiscent
*Hydrochorea* – African continent (sensu Soares et al. (2022))	straight to falcate or slightly curved	papery	yes	loment, seeds dispersed in 1-seeded articles
*Hydrochorea* – Neotropics (sensu Soares et al. (2022))	straight to falcate or slightly curved	papery or ligneous to woody	yes	loment, cryptoloment, follicular or indehiscent
*Albizia* – African continent	straight	papery	no	dehiscent
*Albizia* – Madagascar	straight or twisted	papery, thinly ligneous or woody	variable	dehiscent or indehiscent
*Albizia* – Asia, Australia and Oceania (incl. *Cathormion* s.s.)	straight, slightly curved or coiled/contorted	papery or woody	variable	dehiscent or tardily breaking up into articles
Albiziasect.Arthrosamanea (= *Pseudalbizzia*) – Neotropics	straight	papery, coriaceous or ligneous	variable	dehiscent, indehiscent, loment or cryptoloment
*Samanea* – Neotropics	straight to slightly curved	thick and ligneous and/or fleshy	yes	indehiscent

## Taxonomic treatment

### Identification key to the continental African genera of ingoid mimosoids

(i.e. the ingoid clade sensu Koenen et al. (2020), which, apart from *Senegalia* Raf. and *Faidherbia* A. Chev., equates to all mimosoid genera native to the African continent with stamens fused into a tube):

**Table:** 

1	Plants with prickles, stamens usually free or sometimes connate at base	***Senegalia***
–	Plants usually unarmed, rarely with spinescent shoots or stipular spines, stamens fused into a tube or at least connate at base	**2**
2	Shrubs with elastically dehiscent pods	***Afrocalliandra* E.R. Souza & L.P. Queiroz**
–	Trees or rarely shrubs with indehiscent, non-elastically dehiscent or lomentiform pods	**3**
3	Trees with stipular spines, inflorescence spicate	***Faidherbia***
–	Usually unarmed trees or with spinescent shoots, but never stipular spines, inflorescences capitate	**4**
4	Fruit non-septate, with papery valves, usually dehiscent	***Albizia***
–	Fruit septate, with thin fibrous or thick woody valves, indehiscent or lomentiform	**5**
5	Peripheral flowers of capitulum on pedicels at least 1 mm long, leaves with (1–)2–3 pairs of pinnae, fruit lomentaceous with seeds dispersed as 1-seeded articles	***Hydrochorea***
–	Peripheral flowers on pedicels up to 0.5 mm long, leaves with (3–)5–30(–35) pairs of pinnae, fruit indehiscent or, if lomentiform, only tardily breaking up into articles	***Osodendron***

#### Osodendron

Taxon classificationPlantaeFabalesFabaceae

E.J.M. Koenen gen. nov.

5D4D1D37-6635-5BCD-A201-EDF8BB6ABED6

urn:lsid:ipni.org:names:77303836-1

##### Type.

*Osodendronaltissimum* (Hook. f.) E.J.M. Koenen.

##### Diagnosis.

*Osodendron* differs from *Robrichia* by pods being either straight to twisted or coiled, but not contorted and ear-shaped.

##### Description.

***Trees***, or rarely ***shrubs***, unarmed or sometimes with spine-like projections on twigs or spine-like outgrowths on adventitious roots, resting buds perulate with scales distinctly different from stipules. ***Indumentum*** of usually dense ferruginous pubescence on twigs, petioles, rachis and pinna rachises, stipules, bud scales and peduncles. ***Leaves*** with (3–)5–30(–34) pairs of pinnae, opposite or sometimes (the lowermost pairs) sub-opposite, with a single petiolar nectary usually present, as well as nectaries between at least some of the pinna pairs on abaxial surface of the rachis and often also between the upper 1–many leaflet pairs on the pinna-rachises, the lowermost pair of pinnae usually distinctly shorter than others, pinnae with (7–)13–40(–48) leaflet pairs, one of the two leaflets of the lowermost pair usually reduced to a small paraphyllidium or lacking. ***Inflorescences*** sub-globose capitula, dimorphic, borne on peduncles arising from axillary fascicles, sometimes arranged in short compound pseudoracemes with the leaves suppressed (not fully developing) and caducous as apparent from the presence of leaf scars in the pseudoracemes, these pseudoracemes developing below the foliage. ***Flowers*** sessile or shortly stipitate, 4- or 5-merous, with fused calyx and corolla, androecium consisting of 10–25 stamens that are fused in the lower part to form a staminal tube, pollen compound in 32-celled polyads, central flowers more robust with a broader nectariferous base and longer staminal tube exserted beyond corolla tube. ***Fruits*** septate, indehiscent or tardily breaking up into articles, either lomentiform, twisted and strongly curved to coiled or weakly to not articulate and slightly curved to straight, seeds with a hard testa and open or closed pleurogram.

##### Distribution and habitat.

Three species in tropical Africa, from Senegal in the west to the Democratic Republic of Congo in the east and Zambia and Angola in the south. Typically occurring in rainforest and extending into the savannah zone in gallery forest.

##### Etymology.

The genus is named after “*Oso*”, a food that is prepared in West Africa (Ghana and Nigeria) by fermenting the seeds of the type species *O.altissimum* and grinding them into a protein-rich paste that is subsequently cooked and eaten as either a main food, a delicacy or as a condiment to flavour soups and stews (Popoola et al. 2004; Jolaoso et al. 2014).

##### Notes.

*Osodendron* is closely related to the Central and South American *Robrichia*, which was originally described as a section of *Enterolobium* by Barneby and Grimes (1996) and later segregated as a new genus by Souza et al. (2022). In the phylogeny of Ringelberg et al. (2022), the sampled species of both genera form sister-lineages. In habit, leaves and flowers, the two genera are very similar; however, the numbers of pinnae per leaf and leaflets per pinna and leaflet dimensions show greater variation in *Osodendron* with especially relatively large leaflets in the type species compared to *Robrichia*. The clearest difference between the two genera is to be found in the fruits, where those of *Robrichia* are contorted (or “ear-shaped”) and indehiscent, while those of *Osodondron* are either straight to falcate and indehiscent or twisted to spirally-coiled and lomentiform, only tardily breaking up into articles.

Species of *Osodendron* can be easily distinguished from African *Albizia* species by the sub-opposite lower few pinna-pairs of which the lowermost is usually distinctly shorter (half or two thirds the length of the next few pairs of pinnae), as well as the strong reduction or lack of one of the two leaflets of the lowermost leaflet pair on each pinna (in *Albizia*, the lowermost pair of pinnae is only slightly shorter at most and the lowermost leaflet pair is not reduced or lacking one leaflet). The fruits are also notably different, with the inertly dehiscent pods of continental African *Albizia* spp. never woody nor articulate and always flat and papery (although in several Asian, Australian and Madagascan *Albizia* spp., indehiscent woody or tardily dehiscent articulate pods occur, for example, Fig. 1B; Table 1).

### Identification key to the species of *Osodendron*

**Table:** 

1	Leaves with fewer than 10 pairs of pinnae; fruits coiled or twisted, rarely only curved, flowers with 20–25 stamens	**1. *Osodendronaltissimum***
–	Leaves with more than 10 pairs of pinnae; fruit straight or somewhat curved, but not coiled nor twisted, flowers with 10–14 stamens	**2**
2	Leaves of mature individuals (not those on coppice shoots) lacking nectaries between upper leaflet pairs, fruit a thick woody indehiscent pod, with uniform flat valves, not distinctly swollen over the seeds and rectangular in cross-section – West Africa (Upper Guinea)	**2. *Osodendrondinklagei***
–	Leaves usually with nectaries between the upper 1–3 leaflet pairs, fruit usually a flat sub-lomentiform indehiscent pod or sometimes a thicker woody pod, articulately swollen around the seeds, elliptic to circular in cross-section – Central and East Africa (Lower Guinea, Congolia and gallery forests in adjacent savannahs)	**3. *Osodendronleptophyllum***

#### 1. Osodendron altissimum

Taxon classificationPlantaeFabalesFabaceae

(Hook. f.) E.J.M. Koenen comb. nov.

BA26D0AF-4AC5-5303-B0FC-76FC0B9B17E7

urn:lsid:ipni.org:names:77303837-1

Figs 1A , 2

Pithecellobium altissimum (Hook. f.) Oliv., Fl. Trop. Afr. [Oliver et al.] 2: 364. 1871.
Feuilleea altissima (Hook. f.) Kuntze, Revis. Gen. Pl. 1: 187. 1891.
Pithecellobium stuhlmannii Taub., Pflanzenw. Ost-Afrikas C 193. 1895. Type material: Democratic Republic of Congo, Bataibo bei Duki, 850 m, *Stuhlmann 2773* (holotype: B†), neotype here designated: Democratic Republic of Congo, Irumu, *Bequaert 4887* (BR!).
Albizia passargei Harms, Bot. Jahrb. 26: 253. 1899. Type material: Cameroon, Ngaoundéré, *Passarge 164* (holotype: B†), neotype here designated: Cameroon, Bordure du Meng près des lacs de Boubala, *Letouzey 2562* (P [P03502143] digital image!).
Cathormion altissimum (Hook. f.) Hutch. & Dandy, Fl. W. Trop. Afr. [Hutchinson & Dalziel] 1: 364. 1928; Hutchinson & Dandy in Kew Bull. 1928 (10): 401. 1928.
Arthrosamanea altissima (Hook. f.) G.C.C. Gilbert & Boutique, Bull. Jard. Bot. État Bruxelles 22: 182. 1952.
Inga altissima (Hook.f.) Roberty, Bull. Inst. Franç. Afrique Noire, A. 16: 343. 1954.

##### Basionym.

*Albiziaaltissima* Hook. f. Niger Fl. [W. J. Hooker]. 332. 1849.

##### Type material.

Ghana, Cape Coast, 7/41, *T. Vogel 18* (lectotype here designated from amongst the syntypes: K!); Nigeria, Aboh, 8/41, *T. Vogel 28* (syntype: K!).

##### Description.

***Tree*** or rarely ***shrub***, unarmed or sometimes with blunt spine-like projections on young twigs (W. Hawthorne, personal communication), 5–35 m tall, stem to 40 cm in diameter. Branches covered with many minute round lenticels 0.1–0.2 mm, twigs, stipules, bud scales, all leaf axes and peduncles brown pubescent, often densely so. Foliage consisting of bipinnate leaves with opposite to sub-opposite pinnae with closely-spaced, distinctly discolourous leaflets, slightly wider in the middle with the apical pair distinctly distally projected, in the basal pair often one or both leaflets reduced to paraphyllidia. The inflorescences consisting of sub-spherical capitula emerging in axillary fascicles, often aggregated into pseudoracemes emerging from the perulate resting buds, with the leaves not fully developing and caducous. ***Stipules*** deltoid to linear, 2–8 ×1.0–1.8 mm, caducous, perulae deltoid, 2–4 × 1.8–3 mm. ***Leaves*** with (3–)5–7(–8) pairs of pinnae, apical pairs usually slightly longer and the basal pair sometimes much shorter with fewer leaflets, petiole 1.3–2.5 cm, pulvinate, adaxially shallowly canaliculate and often laterally and abaxially grooved, with a circular to elliptic sessile cupular nectary ca. 0.8–2.1 mm in diameter located ca. mid-petiole, rachis (1.8–)4.5–11(–14.5) cm, adaxially shallowly canaliculate and often laterally and abaxially grooved, with 2–5 circular to elliptic sessile cupular nectaries between or just below the distal pairs of pinnae ca. 0.5 mm in diameter, pinna-rachises (2.4–)3.5–8(–10.2) cm, proximally pulvinate, laterally compressed, with elliptic to circular sessile cupular nectaries of 0.2–0.8 × 0.2–0.5 mm between the upper 1–4(–15) leaflet pairs, pinnae with (7–)13–20(–25) leaflet pairs, very often the abaxial leaflet of the lowermost pair reduced to a paraphyllidium, leaflets sessile, pulvinate, inequilaterally hastate or weakly sigmoid, with acute or sometimes obtuse apex or (in subsp. busiraensis) weakly rhombic with oblique base and mostly obtuse apex or sometimes apiculate, (4–)7–14(–18) × (1.5–)3–4 mm, asymmetrically palmately-pinnate secondary venation with 7–10 veins starting from leaflet base next to the mid-rib and (7–)15–22 major and intercalary secondary veins laterally from the mid-rib, brochidodromous to craspedodromous, distinctly prominent on both surfaces when dry (or obscure on adaxial surface in subsp. busiraensis), tertiary venation finely reticulate, hardly visible on adaxial surface. ***Capitula*** ca. 35–50-flowered, on peduncles 1.8–3.2 cm, dimorphic with 3–9 central flowers with broader base and longer staminal tubes, bracts lanceolate to lanceolate-spatulate, ca. 0.9–1.2 × 0.1–0.2 mm, ciliate in upper half, caducous. Peripheral flowers slenderly campanulate with pale green or greenish-white calyx and corolla, subsessile to shortly pedicellate, pedicel to ca. 0.5 mm, sparsely pubescent. Calyx 5-merous, 2–3.2 mm long, the deltoid lobes ca. 0.5 × 0.5 mm, ciliate at the apex and often also a few scattered short hairs on the outer surface of calyx tube and lobes, otherwise glabrous, corolla 5-merous, 5–7.5 mm long, glabrous, androecium of ca. 20–25 stamens, filaments white, ca. 12–17 mm long, fused into a tube at the base for ca. 2–3 mm, anthers yellow, basifixed, pollen aggregated into 32-celled flattened polyads. Pistil ca. 13–18 mm long, the ovary ca. 3 mm with a few scattered hairs on distal half and the funnel-shaped stigma extending ca. 1 mm beyond the anthers. Central flowers similar, but sessile, broadly campanulate and staminal tube ca. 5.5–8.5 mm long (i.e. the stamens are approximately the same length, but the fused tube is longer than in peripheral flowers), the tube exserted beyond the corolla. ***Fruit*** a spirally-curved, twisted or coiled lomentiform pod, usually articulate between the seeds, breaking up tardily after abscission in water or on the forest floor, often with arenchymous swollen mesocarp for flotation, (10–)18–29(–30) × (0.8–)1.1–1.7 × 0.2–0.6 cm, (3–)9–20(–23) seeded. Seeds plano-compressed, nearly round or slightly elliptic, ca. 5–7.5 × 4–6 × 1.5–2 mm, chestnut brown with a small 2.1–3.2 × 1–1.5 mm elliptic to obovate or oblong open pleurogram.

##### Notes.

A specimen seen at Kew, of *Espírito Santo 1858*, collected in Guinée-Bissau, is doubtfully placed in this species. This specimen is highly unusual in the number of pairs of pinnae (13) and leaflet pairs (14–35) and the leaflets appear relatively small and elongate (even though they appear not fully expanded), while having the typical asymmetrical hastate shape. Furthermore, the fruit is not clearly articulate and also differs in being only somewhat curved rather than twisted or coiled. Brenan and Brummitt (1965) also cited this material as being unusual and speculated that it may be a distinct taxon. I do not share this opinion as I do not think it is sufficiently morphologically distinct. It is perhaps more likely of hybrid origin with *O.dinklagei*, which occurs sympatrically, as the other parent. However, this unusual specimen resembles *O.altissimum* much more closely than *O.dinklagei* and the number of pinnae and leaflets and dimensions of the leaflets clearly distinguish it from that species.

The holotype of *Pithecellobiumstuhlmannii* was destroyed at B and no surviving isotypes are known. According to the Flora of East Tropical Africa (Brenan 1959), the original material was collected in the Democratic Republic of Congo, west of Lake Albert. The specimen of *Bequaert 4887* from BR is from the same region, includes leaves, flowers and immature fruit and matches the description in the protologue and is here selected as the neotype of *P.stuhlmannii*.

The holotype of *Albiziapassargei* was also destroyed at B, but Villiers (1989) placed the name in synonymy with *C.altissimum*, based on the description in the protologue. A specimen of *Letouzey 2562* from P, comprising leaves and flowers, is here chosen as the neotype as it was collected in N Cameroon (ca. 150 km SW of the type locality) and matches the description in the protologue.

### Key to subspecies of *O.altissimum*:

**Table:** 

1	Leaflets typically asymmetrically hastate and weakly sigmoid, apex acute, usually > 3× as long as wide; fruits spirally-curved, coiled or twisted, 1-seeded articles (11–)13–17 mm wide and > (3–)4 mm thick when ripe – widespread in Lower and Upper Guinea	**1.1. Osodendronaltissimumsubsp.altissimum**
–	Leaflets weakly rhombic with oblique base, apex obtuse, ≤ 3× as long as wide; fruits spirally-coiled, 1-seeded articles 8–11 mm wide and 2–3 mm thick when ripe – Local in west Democratic Republic of Congo	**1.2. Osodendronaltissimumsubsp.busiraensis**

#### 1.1 Osodendron altissimum subsp. altissimum

Taxon classificationPlantaeFabalesFabaceae

9FF091AF-2B4D-5B43-954E-3ACE0CEA1992

##### Distribution.

Widespread across Upper and Lower Guinea and Congolia, from Guinea-Bissau in the west to the Democratic Republic of Congo in the east and Angola in the south (Fig. 2A), also reported from Uganda and Zambia (Brenan 1959).

##### Ecology.

Fresh-water swamp forest and along rivers in primary and secondary, evergreen or semi-deciduous rainforest.

##### Representative specimens studied.

**Guinée-Bissau**: *Espírito Santo 3810* & *3832* (frts., BR); **Côte d’Ivoire**: *Oldeman 968* (fls, BR); **Cameroon**: *A.J.M. Leeuwenberg 8868* (fr., BR), *B. Sonké 5499* (fr., BR); **Gabon**: *N. Hallé 2119* (fls, frts, BR); **Democratic Republic of Congo**: *F. Seret 461* (fls, BR);

#### 1.2. Osodendron altissimum subsp. busiraensis

Taxon classificationPlantaeFabalesFabaceae

(G.C.C. Gilbert & Boutique) E.J.M. Koenen, stat. nov. et comb. nov.

A7DA518F-BF2B-555D-B635-58259082E9D1

urn:lsid:ipni.org:names:77303839-1

##### Basionym.

Arthrosamaneaaltissimavar.busiraensis G.C.C. Gilbert & Boutique, Fl. Congo Belge 3: 183. 1952.

##### Type material.

Democratic Republic of Congo, Eala surroundings, Wangata-Watsiko, October 1943, *Germain 1604* (holotype: BR! [BR0000015846686], isotype: P [P00418269] digital image!).

##### Note.

This subspecies was originally described as an infraspecific variety. I have considered treating it as a distinct species, but after studying material held at BR, I concluded it cannot be reliably separated from the typical subspecies across its entire range. The material stands out in its combination of slightly different leaf shape and smaller pods, but material outside the Busira river catchment in the Democratic Republic of Congo, as well as in other parts of Africa, is rather variable in leaf shape and pod size. Given that both these differences are consistently present in most of the material from that region, i.e. the variation is geographically structured within this variable and widespread species, subspecies is more appropriate than varietal rank and I treat it here as such.

##### Distribution.

Local to the Busira river catchment in the western Democratic Republic of Congo (Fig. 2A).

##### Ecology.

As the typical subspecies.

##### Representative specimens studied.

**Democratic Republic of Congo**: *Boyekoli Ebale Congo Expedition 2010 104* (fls, BR); *J. Louis 2052* (BR) & *13196* (BR); *Robyns 653* (BR); *Ghesquiere 2787* (BR); *G. Hulstaert 607* (BR).

#### 2. Osodendron dinklagei

Taxon classificationPlantaeFabalesFabaceae

(Harms) E.J.M. Koenen comb. nov.

B1C0D13B-3EDF-5C32-9DED-71CDBDE5CB09

urn:lsid:ipni.org:names:77303840-1

Fig. 3A–F

Albizia dinklagei (Harms) Harms, Bot. Jahrb. Syst. 53(3–5): 455, in obs. 1915.
Pithecellobium dinklagei (Harms) Harms, Notizbl. Bot. Gart. Berlin-Dahlem 8: 145. 1922.
Cathormion dinklagei (Harms) Hutch. & Dandy, Fl. W. Trop. Afr. [Hutchinson & Dalziel] 1: 364. 1928; Hutchinson & Dandy in Kew Bull. 401. 1928.
Samanea dinklagei (Harms) Keay, Kew Bull. 8(4): 488. 1954.

##### Basionym.

*Mimosadinklagei* Harms, Bot. Jahrb. Syst. 26(3–4): 253. 1899.

##### Type material.

Liberia, Grand Bassa, 21 May 1897, *Dinklage 1827* (holotype: B†, isotype: K! [K000044068]).

##### Description.

***Tree***, up to 30 m tall, twigs with rows of lenticels and becoming shallowly grooved with age. Twigs, stipules, perulate resting buds, all leaf-axes and peduncles covered in a densely villose ferruginous indumentum. Foliage consisting of very finely divided bipinnately compound leaves, the tiny leaflets ciliate and somewhat discolourous. The inflorescences consisting of sub-spherical capitula emerging from axillary fascicles of coeval leaves, sometimes in compound ramiflorous pseudoracemes with the leaves not fully developing and caducous. ***Stipules*** narrowly deltoid or lanceolate to linear 3.5–4.3(–9) × ca. 1 mm, caducous leaving conspicuous glabrous scars, perulae deltoid, ca. 2 × 1.5 mm. ***Leaves*** with 10–34 pairs of pinnae, often with one or a few much reduced pinnae at the base, petiole 5–11 mm, pulvinate and adaxially flattened at the base, usually with a sessile or shortly stipitate cupular nectary 0.5–0.7 mm in diameter just below the lowermost pinna pair, rachis grooved and adaxially canaliculate although obscured by dense indumentum, with a 1–1.5 mm long deciduous mucro, 6.5–16(–21) cm, with 3–8(–11) interpinnal subsessile cupular nectaries apically, these sometimes button-shaped and on a 0.7 mm long stipe, pinnae obscurely pulvinate, with an adaxial ridge and a tiny 0.3–0.4 mm long mucro at the apex, 1.0–2.5(–4.5) cm, usually without nectaries, except on larger leaves (of juveniles or coppice shoots) with cupular nectaries between the upper 1–3 leaflet pairs, pinnae with (9–)27–40(–48) leaflet pairs, the abaxial leaflet of the lowermost pair usually reduced to a paraphyllidium, leaflets inequilaterally linear, sessile and pulvinate, with strongly oblique base and acute apex, 0.8–3 × 0.2–0.8 mm, venation obscure on the adaxial surface, palmately-pinnate brochidodromous, with three secondary veins emerging from the base next to the clearly stronger mid-rib on the adaxial side, 2–3 further secondary veins on each side and sparse reticulate tertiary venation. ***Capitula*** 1–3 per leaf axil, ca. 40–60-flowered, on peduncles 1.8–3.5(–4.1) cm, dimorphic with 1(–3) central flower(s) that are more robust and with a longer, exserted staminal tube. Peripheral flowers ca. 0.7 mm broad at the base, the basal ones on a pedicel of ca. 0.4–0.6 mm, the others sub-sessile to sessile closer to the apex. Calyx slenderly campanulate, green, 5-merous, 1.6–3.1 mm long, densely puberulous on outer surface, the deltoid lobes ca. 0.4 × 0.4 mm, corolla green, 4- or 5-merous, 3–5.5 mm long, pubescent, the lobes 1.8–2 mm long, stamens 10–14, filaments white, 14 mm long, basally fused into a tube ca. 3 mm long, anthers light yellow, basifixed, pollen released in 32-celled polyads, pistil ca. 18 mm long with a sessile ovary ca. 2.5 mm long, puberulent on upper half with white hairs, stigma shallowly funnel-shaped. Central flower(s) similar to peripheral flowers, but ca. 1.8 mm broad at the base, calyx 2.0–2.4 mm long, corolla ca. 4.5 mm long, staminal tube ca. 6 mm long (i.e. the stamens are approximately the same length, but fused for a greater part than in peripheral flowers). ***Fruit*** an indehiscent woody pod up to 13–18 × 2–2.2(–2.5) × 0.3–0.6 cm, straight or slightly curved and with uniformly thick valves, except for the distinctly thickened margins, not swollen over the seeds, 16–24 seeded when well-fertilised, seeds rounded elliptical, only slightly laterally compressed, greenish light brown, 6–7.5 × 4–5 × 3–3.5 mm when dry, with a hard testa and an elongate closed pleurogram 5–6 × 2–2.5 mm.

##### Distribution.

Upper Guinea, from Guinea-Bissau to Ghana (Fig. 3G).

##### Ecology.

Rainforest, gallery forest, wooded grassland, edges of mangrove swamp.

##### Representative specimens examined.

**Guinée-Bissau**: *Espírito Santo 1747* (K) & *1864* (K) & *1871* (K) & *2697* (K). **Sierra Leone**: *X. van der Burgt 1994* (K).

#### 3. Osodendron leptophyllum

Taxon classificationPlantaeFabalesFabaceae

(Harms) E.J.M. Koenen comb. nov.

8280CC22-67BA-5478-9558-3B890922FA3B

urn:lsid:ipni.org:names:77303841-1

Fig. 3H–K

Albizia eriorhachis Harms syn. nov., Bot. Jahrb. Syst. 53(3–5): 456. 1915. Type material: *Chevalier 7777* (lectotype here designated from amongst the syntypes: P [P00418361] digital image!, isolectotypes: P [P00418360] digital image!, P [P00418362] digital image!, L [L.1992190] digital image!).
Albizia flamignii De Wild., Pl. Bequaert. 3: 49. 1925. Type material: Democratic Republic of Congo, Kitobola, 12 Sept 1910, *Flamigni 265* (lectotype designated here: BR! [BR0000016145290], isolectotypes: BR! [BR0000016145283], BR! [BR0000016145306], P [P00418365], digital image!).
Cathormion eriorhachis (Harms) Dandy syn. nov., in F. W. Andrews, Fl. Pl. Anglo-Egypt. Sudan ii. 155. 1952. Type: Based on Albiziaeriorhachis Harms.
Arthrosamanea leptophylla var. guineensis G.C.C. Gilbert & Boutique, Bull. Jard. Bot. État Bruxelles 22: 182. 1952. Type material: Democratic Republic of Congo, Yangambi, réserve flore Isalowe, févr. 1940, *Louis 16368* (BR!).
Samanea guineensis (G.C.C. Gilbert & Boutique) Brenan & Brummitt, Bol. Soc. Brot. sér. 2, 39: 202. 1965. Type: Based on Arthrosamanealeptophyllavar.guineensis G.C.C. Gilbert & Boutique.

##### Basionym.

*Albizialeptophylla* Harms, Bot. Jahrb. Syst. 53(3–5): 455. 1915.

##### Type material.

Democratic Republic of Congo, Kimuenza, 17 km S of Leopoldville, August 1910, *Mildbread 3520* (lectotype (designated by Villiers (1989) as holotype, here corrected): HBG [HBG519160] digital image!, isolectotypes: HBG [HBG519161] digital image!, P [P00418364] digital image!).

##### Description.

***Tree*** or more rarely a ***shrub***, unarmed or with spine-like outgrowths near the base of the bole perhaps associated with adventitious roots (W. Hawthorne, personal communication), to 30 m tall and to 1.25 m in stem diameter, twigs dark with densely scattered small pustular lenticels or sometimes with lighter coloured corky bark. Twigs, stipules, perulate resting buds, all leaf-axes and peduncles covered in dense ferruginous villose indumentum. Foliage consisting of finely divided bipinnate leaves, the leaflets variably ciliate, sometimes with only very few hairs on the margins, the lamina usually glabrous, but sometimes also with a few scattered hairs along the mid-rib on lower surface or rarely pilose or with appressed long hairs on both surfaces especially in lower half, usually distinctly discolourous. The inflorescences consisting of sub-spherical capitula emerging from axillary fascicles of coeval leaves, usually a few below the leaves in the axils of caducous leaves on the same shoots or sometimes also with a few short ramiflorous pseudoracemes, with all leaves caducous, lower down on the branch. ***Stipules*** elliptically oblong to asymetrically oblanceolate or falcate, 5–8(–9) × 2–3 mm, more scarcely pubescent or nearly glabrous on adaxial surface, caducous leaving conspicuous scars, perulae deltoid to ovoid, ca. 1.5–5 × 1.5–3.5 mm. ***Leaves*** with (10–)12–30(–35) pairs of pinnae, the lower 1–2 pairs of pinnae often shorter with fewer leaflets, petiole pulvinate and flattened on adaxial side at the base, (0.5–)0.8–2.2(–3) cm long, petiolar nectary usually present, cupular and circular, sessile or shortly stipitate and 0.6–1 mm in diameter, in variable position ranging from just above the pulvinule to just below the basal pair of pinnae, rachis canaliculate although often obscured due to dense pubescence, 8–15(–22) cm long, with 1–4(–6) cupular nectaries between the upper pairs of pinnae, these 0.3–0.6 mm in diameter, pinnae pulvinate and with an adaxial ridge, (0.5–)2.5–5(–7.5) cm long, nearly always the abaxial leaflet of the lowermost pair reduced to a paraphyllidium and the adaxial one somewhat smaller than the other leaflets, with elliptical nectaries ca. 0.1–0.25 mm in diameter between the upper 1–3 leaflet pairs, pinnae with (17–)24–35(–42) leaflet pairs, leaflets sessile, pulvinate, asymmetrically oblong to oblanceolate, base oblique to sometimes hastate, apex rounded to acute, (2–)3–6(–9) × (0.3–)0.5–1.2(–2) mm, venation palmately-pinnate brochidodromous, with 2–4 basal veins adaxially next to the mid-rib and 5–8 further secondary veins on each side and reticulate tertiary venation, apart from the mid-rib, all venation often obscure in smaller leaves, otherwise the secondary venation prominent, sometimes only on the lower surface. ***Capitula*** 2–4 arranged in fascicles, on peduncles ca. 2.7–4.5 cm, dimorphic with ca. 50–70 peripheral flowers and a single elongated central flower. Bracteoles spatulate, ca. 0.5 mm long, pubescent on outer surface. Flowers white, the peripheral ones on pedicels 0.75–2.5 mm. Calyx slenderly campanulate, green, 5-merous, 2–2.5 mm long, densely puberulous on outer surface, the deltoid lobes ca. 0.25 mm long, corolla 4–4.5 mm long, pubescent on outer surface, the lobes 1.8–2 mm long, stamens 10–14, the filaments white, 11–14 mm long, basally fused into a tube 1.5–2.5 mm long, anthers basifixed, pollen released in 32-celled polyads, pistil on a ca. 0.25–0.75(–1.25) mm long stipe, ovary ca. 1–1.5 mm long, pubescent, style 12–16 mm long, stigma shallowly funnel-shaped. Central flower(s) similar to peripheral flowers, but sessile and longer, calyx ca. 2.5 mm long, corolla ca. 6–6.5 mm long, staminal tube 4–6 mm long, exserted beyond corolla tube. ***Fruit*** a dark brown to black indehiscent pod that may tardily disintegrate into 1–multiple-seeded parts, the slightly thickened margins usually straight or also often articulate especially around aborted seeds, the valves papery and thin or with somewhat thickened mesocarp, but not really ligneous, when ripe, swollen over the seeds, when well-fertilised, 12–28(–32)-seeded, (4.5–)10–16(–20) × 0.9–1.3 × 0.4–0.7 cm. Seeds yellowish-brown, 5.5–7.5 × 3–4.5 × 2–3 mm with hard testa and a darker closed pleurogram of 4.5–5.5 × 1.2–1.5 mm.

##### Distribution.

Lower Guinea and Congolia in Cameroon, Central African Republic, Gabon, Republic of the Congo, Democratic Republic of Congo, Angola (Fig. 3G).

##### Ecology.

Forest edges and gallery forest.

##### Representative specimens examined.

**Cameroon**: *J. Mildbread 8584* (K); **Democratic Republic of Congo**: Luidi, Thysville, Lusolo, 16 October 1959, *P. Compere 610* (BR), Isangi, 21 February 1950, *Callens 2363* (BR), Petite vallées d’affluente de la Belanzovi, près son confluent avec la Lubimbe, 26 January 1949, *A. Michelson 877* (BR, K).

##### Notes.

*Osodendronleptophylla* is highly variable in number of pinnae per leaf, number and size of leaflets and density and length of the indumentum. No consistent correlations between any of these variations have been found suggesting that the material referred to two heterotypic synonyms is best included under *O.leptophyllum*. The first of these is *Albiziaeriorhachis*, described by Harms in the same publication as *A.leptophylla*. Specimens from Cameroon, Central African Republic and Gabon, identified as *Cathormioneriorhachis*, appear to be similar to typical *O.leptophyllum*, but with relatively large leaflets and denser indumentum. However, the material is not distinct enough to merit recognition as a separate species, given the large variation in leaf dimensions and indumentum in *O.leptophyllum* across its range and is here placed in its synonymy.

The second is *Samaneaguineensis*, which was originally described as a variety of *Arthrosamanealeptophylla*, based on more numerous pairs of pinnae and leaflets per leaf, but Brenan and Brummitt (1965) found it to be distinctive enough to recognise it at species rank. Apart from the difference in foliage, they noted differences in the indumentum of the calyx and corolla (glabrous to subglabrous, instead of densely puberulent) and a longer exserted staminal tube. I have observed material that fits their observations (e.g. *Jean Louis 10051*, BR), but in other collections with the leaf type of “guineensis”, I have observed dense pubescence on the calyx (e.g. *Michelson 877*, BR) and vice versa specimens with ca. 15 pairs of pinnae and a subglabrous calyx (*Letouzey 9816*). Villiers (1989) also judged *S.guineensis* to be conspecific with *S.leptophylla*, having also seen numerous specimens with intermediate foliage. I follow Villiers (1989) and place this name in the synonymy of *O.leptophyllum*.

## Supplementary Material

XML Treatment for Osodendron

XML Treatment for Osodendron altissimum

XML Treatment for Osodendron altissimum subsp. altissimum

XML Treatment for Osodendron altissimum subsp. busiraensis

XML Treatment for Osodendron dinklagei

XML Treatment for Osodendron leptophyllum
